# Evaluation of the Cytotoxicity of Two Types of Triple Antibiotic Paste on Human Permanent Dental Apical Papilla Stem Cells: an *in vitro*in vitro Study

**DOI:** 10.30476/DENTJODS.2021.89588.1422

**Published:** 2022-06

**Authors:** Rezvan Rafatjou, Arghavan Kamali Sabeti, Bahar Ahmadi, Sara Soleimani Asl, Maryam Farhadian

**Affiliations:** 1 Pedodontist, Dept. of Pediatric Dentistry, Dental School, Hamedan University of Medical Sciences, Hamedan, Iran; 2 Dentist, Resident of Pediatric Dentistry, Dental School, Hamedan University of Medical Sciences, Hamedan, Iran; 3 Dept. of Anatomy, School of Medicine, Hamadan University of Medical Sciences, Hamadan, Iran; 4 Dept. of Biostatistics, Hamadan University of Medical Sciences, Hamadan, Iran

**Keywords:** Toxicity, Stem cells, Antibiotic complex, Tooth Apex

## Abstract

**Statement of the Problem:**

The use of a new antimicrobial combination in the regenerative endodontic treatment of immature teeth pulp necrosis is a well-known method. Concerns have been raised about the destructive effect of this combination on the stem cells from the apical papilla of permanent human teeth, and there is a study gap.

**Purpose:**

The main objective of the present study was to investigate the cytotoxic effect of modified triple antibiotic paste (mTAP) on stem cells from the apical papilla (SCAPs) of permanent human teeth.

**Materials and Method:**

In this *in vitro* study, stem cells were removed from the immature teeth. After cultivation and third passage, metronidazole, ciprofloxacin, minocycline, and clindamycin were placed in the cell culture medium alone , paired, and in combinations as triple antibiotic paste (TAP) (metronidazole, ciprofloxacin, and minocycline) and mTAP (metronidazole, ciprofloxacin, clindamycin) with doses of 25, 50, 100, 200, 400μg/ml. After 1 and 3 days, cell viability in the culture medium was assessed using the MTT method ([4,5-dimethylthiazol-2-yl]-2,5-diphenyltetrazolium bromide). SPSS software version 24, descriptive statistics methods, and statistical tests such as Kruskal-Wallis and Mann-Whitney tests were adopted to analyze the data.

**Results:**

Analysis of MTT findings indicated that the use of mTAP at 100μg/ml and TAP at 200μg/ml had no adverse cytotoxic effect on stem cells in the first 24 hours, compared to the control group. The cell viability decreased at higher concentrations, although it was not statistically significant. After 72 hours, the toxicity of concentrations higher than 100μg/ml of mTAP and 400 μg/ml of TAP significantly mitigated the percentage of viable cells.

**Conclusion:**

The obtained results demonstrated that the concentration of 100 μg/ml of mTAP could replace TAP in regenerative endodontic treatments at the studied time intervals without worrying about the toxicity.

## Introduction

Endodontic regenerative methods are biologically based on methods, which are devised to replace damaged tissues such as dentin and root structure and dentin-pulp complex cells [ 1 ]. Since the time that the protocol proposed by Banchs and Trope (2004) [ 2 ], several studies have focused on the treatment of immature permanent teeth regarding the clinical efficacy of methods and materials to enhance the regenerative endodontic outcome protocol. Given that the reduction of microbial load in regenerative endodontics is realized by in-channel washing and dressing, the selection of a proper material might be a critical factor for attaining effective cavity disinfection, and the balance between the antimicrobial effect of chemicals and their harmlessness to stem cells is of great importance [ 3 ]. In 1996, Hoshino *et al.* [ 4 ] proposed a combination of metronidazole, ciprofloxacin, and minocycline, called triple antibiotic paste (TAP), as an alternative to calcium hydroxide for intracanal dressing in the regenerative process, due to the induction of necrotic tissue by calcium hydroxide. Due to the multimicrobial nature of dental infections, the single use of antibiotics cannot create a medium, free from bacteria in the root canal. As a result, using a combination of antibiotics against all endodontic pathogens is required to hinder the microbial resistance [ 5 ].

Both in *in vitro* [ 6 ] and in clinical trials [ 7 - 8 ], various antibiotic combinations such as TAP have been shown to be highly efficient against common bacteria in the root canal system, Numerous studies have reported that minocycline causes discoloration of teeth [ 4 , 7 - 8 ], and many attempts have been made to reduce the discoloration when using TAP [ 9 ]. However, this antibiotic is difficult to be obtained in the Iranian market. Unlike minocycline, clindamycin does not discolor deciduous teeth and the permanent teeth replacing them and it is readily available. Furthermore, clindamycin has fewer side effects and drug interactions than erythromycin and amoxicillin, and penetrates well into the most tissues and abscesses, and its long half-life is effective on anaerobic gram-positive and gram-negative bacteria [ 10 ]. Casamassimo *et al.* [ 11 ] recommended that minocycline should be replaced with clindamycin in TAP. The new combination was named modified triple antibiotic paste (mTAP). Recent studies have shown successful results using TAP and mTAP in the treatment of deciduous tooth pulp, and the results of antimicrobial tests of both compounds have been quite similar [ 12 ]. In addition, Lin *et al.* [ 13 ] showed in a study that regenerative endodontic treatment using other mix paste in immature permanent teeth with apical periodontitis causes disappearance of periapical lesions in all specimens after 12 months of follow-up. The cytotoxic effect of TAP on primary pulp stem cells of deciduous teeth has also been reported [ 14 ]. There are concerns about the destructive effect of mTAP on the stem cells from the apical papilla (SCAPs) of immature permanent teeth. Therefore, this study aims to investigate the cytotoxicity of the mTAP complex on the SCAPs of permanent human teeth in comparison with TAP.

## Materials and Method

### Sample selection

After obtaining informed consent from the patients, four healthy first immature premolars with open apex (more than 1.5 mm diameter) were selected from two healthy patients referred to the Pediatric Department of Dental School with the age range of 8-10 years with no history of systemic diseases to extract SCAPs. In their orthodontic treatment plan, extractions of these premolars were indicated. Two to five days before tooth extraction, the patient underwent complete dental health training and prophylaxis, and on the day of extraction, the patients received tooth prophylaxis as well. Before and after anesthesia injection, patients rinsed their mouth with 0.2% chlorhexidine mouthwash once for 30 seconds.

### Harvesting of SCAP and Cell Culture

**Figure 1:**
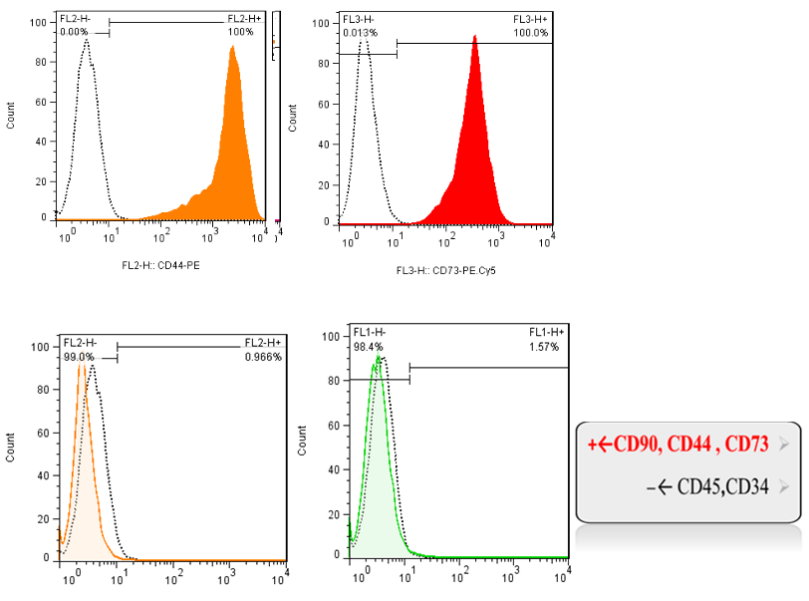
Flow cytometric analysis of stem cells from the apical papilla (SCAPs)

The teeth were extracted with sterile instruments and were immediately placed in a warm sterile PBS [Gibco, USA, Idehzist] solution containing 1% penicillin and streptomycin . SCAPs were isolated From the apical papilla tissue of incompletely developed tooth using Dental tweezer and placed in a digestive solution containing 1% penicillin-streptomycin and 3 mg/ml collagenase type I[Sigma, Germany, then they were cultured in α-MEM [Gibco, USA, Idehzist] containing 1% penicillin (to prevent the growth of gram-positive bacteria) and streptomycin (to prevent the growth of gram-negative bacteria) and 20% fetal bovine serum (FBS) [Sigma Aldrich; Germany] [ 15 ]. The cell culture medium was changed every two days. Fungisome (Amphotericin B) (2μg/l) was used to avoid fungal infection in the primary dental culture [ 16 ]. Cell passage was performed after cell density in cell colonies reached about 80-70%. Third passage cells were used to assess the cytotoxicity of the drugs. Flow cytometric analysis was performed in the third passage to evaluate the nature of SCAPs and the expression of surface markers (Figure 1).

### Preparation of antibiotics

First, the necessary medications, including metronidazole (5g), ciprofloxacin (5g), minocycline (25mg) and clindamycin (1g) (Sigma Aldrich; Germany), were prepared, and weighed by a digital scale with an accuracy of 0.000g. Then 25, 50, 100, 200, 400µg/ml of each medication were prepared, and equal proportions of each medication in the prepared concentration were mixed for TAP and mTAP [ 12 ]. It should be noted that the solvent of antibiotics was cell culture medium. The prepared medications were added to cell culture plates, and 24 and 72 hours later, cell viability in the culture medium was evaluated using 3-(4,5-dimethylthiazol-2-yl)-2,5-diphenyl tetrazolium bromide (MTT) method. 

### Grouping

Group 1: SCAPs were exposed to a metronidazole at doses of 25, 50,100,200,400μg/ml over a period of 24 and 72 hours 

Group 2: SCAPs were exposed to a clindamycin at doses of 25, 50,100,200,400μg/ml over a period of 24 and 72 hours

Group 3: SCAPs were exposed to a minocycline at doses of 25, 50,100,200,400μg/ml over a period of 24 and 72 hours

Group 4: SCAPs were exposed to a ciprofloxacin at doses of 25, 50,100,200,400μg/ml over a period of 24 and 72 hours

Group 5: SCAPs were exposed to a combination of the antibiotics clindamycin and metronidazole at doses of 25, 50,100,200,400μg/ml over a period of 24 and 72 hours

Group 6: SCAPs were exposed to a combination of the antibiotics metronidazole and minocycline at doses of 25, 50,100,200,400μg/ml over a period of 24 and 72 hours

Group 7: SCAPs were exposed to a combination of the antibiotics metronidazole and ciprofloxacin at doses of 25, 50,100,200,400μg/ml over a period of 24 and 72 hours

Group 8: SCAPs were exposed to a combination of the antibiotics clindamycin and ciprofloxacin at doses of 25, 50,100,200,400μg/ml over a period of 24 and 72 hours

Group 9: SCAPs were exposed to a combination of the antibiotics minocycline and ciprofloxacin at doses of 25, 50,100,200,400μg/ml over a period of 24 and 72 hours

Group 10: SCAPs were exposed to a combination of mTAP at doses of 25, 50,100,200,400μg/ml over a period of 24 and 72 hours

Group 11: SCAPs were exposed to a combination of TAP at doses of 25, 50,100,200,400μg/ml over a period of 24 and 72 hours

### Data analysis method

After calculating the percentage of living cells, the amount of cell toxicity was defined as High toxicity (less than 30% of cells survive), Moderate toxicity (30-60% of cells survive), Toxicity at least (90-60% of cells survive), and No toxicity (more than 90% of cells survived) [ 14 ].

The results obtained from each concentration were compared with the control group, considering the rate of 100% cell viability for the control group, and were reported as a percentage. Values ​​greater than one hundred indicated cell growth and values ​​less than one hundred indicated cell death. Using the measurement of cell viability by MTT (Sigma Aldrich; Germany) and ELISA reader (Rayto, RT-2100C), the results were collected, and study data were obtained. The experiment was carried out in triplicate. That is, three wells were added from each concentration of the drug. In addition to wells containing drugs, the well was used without the addition of drugs to control the testing process. They were analyzed using SPSS statistical software (version 24), descriptive statistics methods and statistical tests such as Kruskal-Wallis and Mann-Whitney tests. The significance level in all tests was equal to 0.05.

## Results

**Figure 2:**
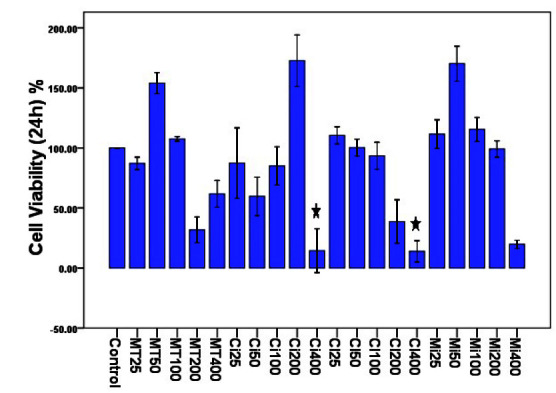
Distribution of cell viability after application of single antibiotics (metronidazole, ciprofloxacin, clindamycin, minocycline) in different doses (25, 50, 100, 200, 400µg/ml) after 24 hours

**Figure 3:**
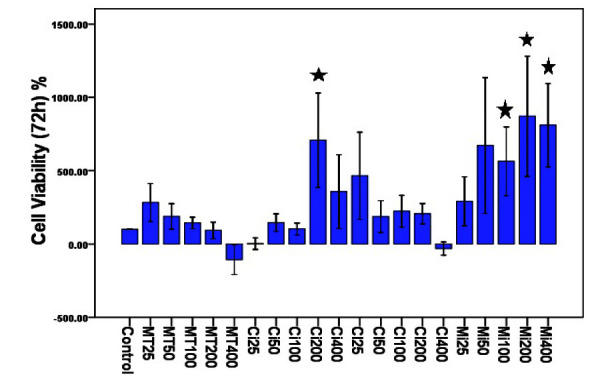
Distribution of cell viability after application of single antibiotics (metronidazole, ciprofloxacin, clindamycin, minocycline) in different doses (25, 50, 100, 200, 400µg/ml) after 72 hours

Figures 2 and Figures 3 and Table 1 depict the results of the investigation of cell viability after applying different doses and combinations of drugs at 24 and 72 hours.

**Table 1:** Mean values of cell viability after applying ternary combinations of antibiotics (TAP & mTAP) in different doses during 24 and 72 hours

Antibiotic	24h			72h			*p* Value* (comparison with control)	
	Mean±SD	Min	Max	Mean±SD	Min	Max	24h	72h
Control	100.00±.00	100.00	100.00	100.00±.00	100.00	100.00		
TAP 25	127.35±19.82	110.93	149.38	81.98±22.60	65.77	107.81	.291	.735
TAP 50	114.22±38.71	78.24	155.19	104.94±16.69	89.50	122.66	.720	.899
TAP 100	129.78±42.84	96.91	178.24	68.08±55.01	13.42	123.44	.435	.460
TAP 200	134.12±40.22	99.45	178.24	77.18±40.37	48.99	123.44	.342	.435
TAP 400	80.31±17.36	61.18	95.06	4.88±29.80	-23.49	35.94	.311	.013*
Other mix 25	112.86±30.28	78.24	134.43	30.98±34.66	-5.47	63.54	.540	.057
Other mix 50	107.72±16.36	92.94	125.31	46.82±30.78	11.41	67.19	.767	.139
Other mix 100	98.56±29.90	64.12	117.90	64.25±27.32	37.58	92.19	.966	.291
Other mix 200	72.36±10.89	62.35	82.51	22.09±39.79	-20.81	57.81	.205	.028*
Other mix 400	50.71±10.89	41.76	62.84	-40.15±21.72	-63.09	-19.89	.069	.002*
*p* Value	.049			.014			*Bonferroni correction	

Triple antibiotic paste (TAP) (metronidazole, ciprofloxacin, and minocycline). mTAP (metronidazole, ciprofloxacin, clindamycin)

When applying ciprofloxacin, a concentration of 400 μg/ml of ciprofloxacin significantly reduced the cell viability in the first 24 hours (compared to the control group) 12 times more than 200μg/ml.

With clindamycin, after 24 hours, a concentration of 400μg/ml led to a significant 80% reduction in cell survival, compared to 100 μg/ml, and after 72 hours, up to a concentration of 200 μg/ml, the cell survival rate was higher, but at a concentration of 400 μg/ml, a significant reduction in cell survival was observed. After 72 hours, minocycline increased cell survival by increasing the antibiotic concentration, which was statistically significant at 100, 200, and 400μg/ml.

The TAP combination did not exhibit an apparent cytotoxic effect in the first 24 hours up to the concentration of g 200 µg/ml, compared to the control group, but at the concentration of 400 µg/ml, the percentage of cell viability decreased by 50% compared to the concentration of 200 µg/ml. In addition, after 72 hours, the concentration of 100 µg/ml showed a 40% reduction in cell viability compared to 50 µg/ml. There is a statistically significant difference in the 400 µg/ml concentration of TAP after 72 hours (Table 1).

In the case of the mTAP combination, the concentration of 100 µg/ml showed a 14% reduction compared to 200 µg/ml for cell survival during the first 24 hours, and 50% of the cells were destroyed at a concentration of 400 µg/ml compared to the control group. After 72 hours, cell growth increased up to the concentration of 100 µg/ml, but at the concentration of 400 µg/ml, cell survival percentage undergone a negative trend, and the highest cytotoxicity was observed, which was statistically significant (Table 1).

The Kruskal-Wallis test results showed that the difference between the mean cell viability in the groups of single, binary, and ternary antibiotics at different doses at the studied time intervals was statistically significant (*p*< 0.05). Comparing the toxicity of mTAP and TAP on SCAPs at different doses at 24 and 72 hours using the Mann-Whitney test showed that mTAP at 100 µg/ml had similar cytotoxicity to TAP, and TAP at 25 and 50µg/ml (after 72 hours) and 200µg/ml (after 24 hours) is less toxic than mTAP (*p* <0.05).

## Discussion

Endodontic treatment of immature teeth due to caries or trauma has been reviewed since the release of regenerative endodontic procedures. Important studies have been conducted in this field to evaluate the antimicrobial effect and biocompatibility of intracanal medications in regenerative endodontics [ 14 , 17 - 20 ]. Due to the essential need for the survival of SCAPs to promote hard tissue deposition, the present study was performed to determine the toxicity of the two antibiotic combinations, TAP, and mTAP, on SCAPs of permanent human teeth. One of the substances whose benefits in treating pulp diseases of immature permanent teeth have recently been proven is TAP [ 21 - 23 ]. Recent findings by Karczewski *et al.*’s study [ 24 ] suggest using clindamycin-modified antibiotic polymer (polydioxanone) nanofibers as a reliable therapeutic alternative for pastes containing minocycline.

Recently, a cytotoxic effect of TAP on SCAPs at the 50μg/ml concentration has been observed, but such effect was absent at the concentration of 10 μg/ml after 3 and 5 days [ 25 ]. This finding contradicts the findings of the present study. The contradiction may be due to differences in the time intervals used in studies. Although this difference in results may be due to differences in the cells types or manufacturers of the antibiotics acquired by the researchers, clinicians should be aware that antibiotics from different sources can have varying cytotoxic effects. The study by Trevino *et al.* [ 26 ] was the first to demonstrate the effect of detergents on SCAPs cells and highlighted the need to evaluate the effect of each of the chemicals used in regenerative endodontics on SCAPs, along with their known antimicrobial properties. In this regard, Ruparel *et al.* [ 17 ] showed that the clinically used concentrations of TAP, double antibiotic paste (DAP), mTAP, and Augmentin have destructive effects on the survival of SCAPs cells, observing less than 20% of cell survival when using concentrations of 10 mg/mL and 100mg/mL of all four drugs. They deduced that high concentrations of antibiotics have a detrimental effect on SCAPs' survival, while low concentrations are as beneficial as calcium hydroxide for the survival and proliferation. In our study, an increase in the drug concentration led to an increase in cytotoxicity.

In a study, Khoshkhoo Nejad *et al.* [ 27 ] identified TAP as the safest drug for SCAPs, while Augmentin showed some destructive effects. It should be noted that in this study, antibiotic combinations TAP, DAP, and mTAP were used at the dose of 10 mg/mL, which is the minimum inhibitory concentration (MIC). Concerns about stem cell safety have led to a tendency to use lower concentrations of intracanal drugs [ 14 , 17 , 27 ]. Since low doses of antibiotics have been used to make antibiotic-releasing scaffolds [ 28 - 33 ] and due to the suitability of the slow release of low-dose drugs from the scaffold, the study of the toxicity of low-dose antibiotics on different types of stem cells, as well as their antimicrobial effects against different types of bacteria that may help in the construction of scaffolds is essential.

One the important finding of the present research is the similar toxicity effect of TAP and mTAP at a 100 μg/mL concentration at the studied time intervals on SCAPs. In this study, cell growth of SCAPs in binary combinations of antibiotics at low concentrations (25 and 50 μg/mL) was also observed. These findings may show similar behavior of antibiotics at lower concentrations while having different effects at higher concentrations. For the future studies, it is recommended to evaluate the effect of TAP and mTAP at different concentrations at times longer than 72 hours. According to the preliminary results, it seems that the proliferation of apical papilla stem cells can neutralize the primary cytotoxic effect.

## Conclusion

Apical papillary mesenchymal stem cells are present at the root end of immature teeth. The presence of mTAP at a concentration of 100μg/mL can replace TAP at the studied time intervals in regenerative endodontic treatments without any concern about the toxicity.

## Acknowledgement

We thank Hamadan University of Medical Sciences to provide the funding of this research.

## Conflict of Interest:

None declared.

## References

[1] Phumpatrakom P, Srisuwan T ( 2014). Regenerative capacity of human dental pulp and apical papilla cells after treatment with a 3-antibiotic mixture. Endod.

[2] Banchs F, Trope M ( 2004). Revascularization of immature permanent teeth with apical periodontitis: new treatment protocol?. Endod.

[3] Lovelace TW, Henry MA, Hargreaves KM, Diogenes A ( 2011). Evaluation of the delivery of mesenchymal stem cells into the root canal space of necrotic immature teeth after clinical regenerative endodontic procedure. Endod.

[4] Hoshino E, Kurihara Ando N, Sato I, Uematsu H, Sato M, Kota K, et al ( 1996). In vitro antibacterial susceptibility of bacteria taken from infected root dentine to a mixture of ciprofloxacin, metronidazole and minocycline. Int Endod.

[5] Segura Egea JJ, Velasco Ortega E, Torres Lagares D, Velasco Ponferrada MC, Monsalve Guil L, Llamas Carreras JM ( 2010). Pattern of antibiotic prescription in the management of endodontic infections amongst Spanish oral surgeons. Int Endod J.

[6] Sato I, Ando Kurihara N, Kota K, Iwaku M, Hoshino E ( 1996 ). Sterilization of infected rootcanal dentine by topical application of a mixture of ciprofloxacin, metronidazole and minocycline in situ. Int Endod J.

[7] Kim H, Kim Y, Shin S, Park W, Ung IY ( 2010). Tooth discoloration of immature permanent incisor associated with triple antibiotic therapy: a case report. Endod.

[8] Reynolds K, Ohnson D, Cohenca N ( 2009). Pulp revascularization of nerotic bilateral bicuspids using a modified novel technique to eliminate potential coronal discolouration: a case report. Int Endod.

[9] Kim B, Song MJ, Shin SJ, Park JW ( 2012). Prevention of tooth discoloration associated with triple antibiotics. Restor Dent Endod.

[10] Zargar N, Rayat Hosein Abadi M, Sabeti M, Yadegari Z, Akbarzadeh Baghban A, Dianat O ( 2019). Antimicrobial efficacy of clindamycin and triple antibiotic paste as root canal medicaments on tubular infection: an in vitro study. Australian Endo J.

[11] Casamassimo PS, Fields Jr HW, McTigue DJ, Nowak A ( 2013). Pediatric dentistry: infancy through adolescence.

[12] Rafatjou R, Yousefimashouf R, Farhadian M, Afzalsoltani S ( 2019). Evaluation of the Antimicrobial Efficacy of two Combinations of drugs on bacteria taken from infected primary teeth: An in vitro Study. J Eur Arch Paed Dent.

[13] Lin J, Zeng Q, Wei X, Zhao W, Cui M, Gu J, et al ( 2017). Regenerative endodontics versus apexification in immature permanent teeth with apical periodontitis: a prospective randomized controlled study. J Endo.

[14] Chuensombat S, Khemaleelakul S, Chattipakorn S, Srisuwan T ( 2013). Cytotoxic effects and antibacterial efficacy of a 3-antibiotic combination: an in vitro study. J Endo.

[15] Rafatou R, Amiri I, Aneshin A ( 2018). Effect of Calcium-enriched Mixture (CEM) cement on increasing mineralization in stem cells from the dental pulps of human exfoliated deciduous teeth. J Dent Res Dent Clin Dent Pros.

[16] Masoum T, Amiri I, Rafatou R ( 2013). Effect of chitosan on osteogenic properties of mesenchymal stem cell of exfoliated deciduous teeth. J Dent Med.

[17] Ruparel NB, Teixeira FB, Ferraz CC, Diogenes A ( 2012). Direct effect of intracanal medicaments on survival of stem cells of the apical papilla. Endod.

[18] Saberi EA, Karkehabadi H, Mollashahi NF ( 2016). Cytotoxicity of Various Endodontic Materials on Stem Cells of Human Apical Papilla. Iranian Endo J.

[19] Phumpatrakom P, Srisuwan T ( 2014). Regenerative capacity of human dental pulp and apical papilla cells after treatment with a 3-antibiotic mixture. Endod.

[20] Kitikuson P, Srisuwan T ( 2016). Attachment ability of human apical papilla cells to root dentin surfaces treated with either 3Mix or calcium hydroxide. Endod.

[21] Banchs F, Trope M ( 2004). Revascularization of immature permanent teeth with apical periodontitis: new treatment protocol?. Endod.

[22] Diogenes A, Ruparel NB, Shiloah Y, Hargreaves KM ( 2016). Regenerative endodontics: a way forward. J Am Dent Assoc.

[23] Chan EK, Desmeules M, Cielecki M, Dabbagh B, dos Santos BF ( 2017). Longitudinal cohort study of regenerative endodontic treatment for immature necrotic permanent teeth. J Endo.

[24] Karczewski A, Feitosa SA, Hamer EI, Pankajakshan D, Gregory RL, Spolnik KJ, et al ( 2018). Clindamycin-modified Triple Antibiotic Nanofibers: A Stain-free Antimicrobial Intracanal Drug Delivery System. J Endod.

[25] Bi J, Liu Y, Liu XM, Jiang LM, Chen X ( 2018). iRoot FM exerts an antibacterial effect on Porphyromonas endodontalis and improves the properties of stem cells from the apical papilla. Int Endod J.

[26] Trevino EG, Patwardhan AN, Henry MA, Perry G, Dybdal Hargreaves N, Hargreaves KM, et al ( 2011). Effect of irrigants on the survival of human stem cells of the apical papilla in a platelet-rich plasma scaffold in human root tips. J Endod.

[27] Khoshkhounejad M, Sobhi Afshar M, Jabalameli F, Emaneini M, Sharifian M ( 2019). Cytotoxicity Evaluation of Minimum Antibacterial Values of Different Medicaments Used in Endodontic Regenerative Procedures. Eur J Dent.

[28] Althumairy RI, Teixeira FB, Diogenes A ( 2014). Effect of dentin conditioning with intracanal medicaments on survival of stem cells of apical papilla. Endod.

[29] Labban N, Yassen GH, Windsor L, Platt A ( 2014). The direct cytotoxic effects of medicaments used in endodontic regeneration on human dental pulp cells. Dent Traumatol.

[30] Sabrah AH, Yassen GH, Liu WC, Goebel WS, Gregory RL, Platt A ( 2015). The effect of diluted triple and double antibiotic pastes on dental pulp stem cells and established Enterococcus faecalis biofilm. Clin Oral Investig.

[31] Alghilan MA, Windsor LJ, Palasuk J, Yassen GH ( 2017). Attachment and proliferation of dental pulp stem cells on dentine treated with different regenerative endodontic protocols. Int Endod J.

[32] Kamocki K, Nor E, Bottino MC ( 2015). Effects of ciprofloxacin-containing antimicrobial scaffolds on dental pulp stem cell viability in vitro studies. Arch Oral Biol.

[33] Albuquerque MT, Evans D, Gregory RL, Valera MC, Bottino MC ( Clin Oral Investig 2016). Antibacterial TAP-mimic electrospun polymer scaffold: effects on P. gingivalis-infected dentin biofilm.

